# Histologic tissue components provide major cues for machine learning-based prostate cancer detection and grading on prostatectomy specimens

**DOI:** 10.1038/s41598-020-66849-2

**Published:** 2020-06-18

**Authors:** Wenchao Han, Carol Johnson, Mena Gaed, José A. Gómez, Madeleine Moussa, Joseph L. Chin, Stephen Pautler, Glenn S. Bauman, Aaron D. Ward

**Affiliations:** 1 Baines Imaging Research Laboratory, London Regional Cancer Program, London, Ontario Canada; 2 Department of Medical Biophysics, University of Western Ontario, London, Ontario Canada; 3 Department of Pathology and Laboratory Medicine, University of Western Ontario, London, Ontario Canada; 4 Department of Surgery, University of Western Ontario, London, Ontario Canada; 5 Department of Oncology, University of Western Ontario, London, Ontario Canada; 6 Lawson Health Research Institute, London, Ontario Canada

**Keywords:** Cancer imaging, Prostate cancer, Translational research, Prostate, Software

## Abstract

Automatically detecting and grading cancerous regions on radical prostatectomy (RP) sections facilitates graphical and quantitative pathology reporting, potentially benefitting post-surgery prognosis, recurrence prediction, and treatment planning after RP. Promising results for detecting and grading prostate cancer on digital histopathology images have been reported using machine learning techniques. However, the importance and applicability of those methods have not been fully investigated. We computed three-class tissue component maps (TCMs) from the images, where each pixel was labeled as nuclei, lumina, or other. We applied seven different machine learning approaches: three non-deep learning classifiers with features extracted from TCMs, and four deep learning, using transfer learning with the 1) TCMs, 2) nuclei maps, 3) lumina maps, and 4) raw images for cancer detection and grading on whole-mount RP tissue sections. We performed leave-one-patient-out cross-validation against expert annotations using 286 whole-slide images from 68 patients. For both cancer detection and grading, transfer learning using TCMs performed best. Transfer learning using nuclei maps yielded slightly inferior overall performance, but the best performance for classifying higher-grade cancer. This suggests that 3-class TCMs provide the major cues for cancer detection and grading primarily using nucleus features, which are the most important information for identifying higher-grade cancer.

## Introduction

The most used treatment for prostate-cancer (PCa) that is organ-confined is radical prostatectomy (RP), the removal of the prostate gland. Approximately 40% of prostate cancer patients undergo this surgery each year in the United States^1^. Serum prostate-specific antigen (PSA) relapse occurs in 17%–29% of patients, reflecting cancer recurrence^2,3^. Post-surgery prognosis, recurrence prediction, and selection and guidance for adjuvant therapy are all informed by the surgical pathology report. Typical pathology reports include tumor size, location, spread, and aggressiveness levels. In addition, PCa patients are grouped based on the Gleason score (GS), which is computed as the sum of the primary and secondary Gleason grades^3^ at RP, into grade group 1 (GS 6; G3 + 3), grade group 2 (GS 7; G3 + 4), grade group 3 (GS7; G4 + 3), grade group 4 (GS 8; G4 + 4) and grade group 5 (GS 9–10; G4 + 5, G5 + 4, and G5 + 5) disease^4,5^, with treatment determined according to the risk level^6^. Thus, although accurate post-RP risk stratification is crucial, currently, clinical pathology reporting is primarily qualitative and subject to intra- and inter-observer variability. This leads to challenges for quantitative and repeatable pathology reporting and interpretation regarding the lesion size, location, spread, and Gleason grade or score^3,7–10^.

Whole-mount tissue sections, where the entire cross section of tissue from the gross section is mounted to the slide, give the pathologist a better overview to facilitate the identification of multiple tumor foci^11^. If cancerous regions of interest (ROI) could be accurately and precisely contoured on whole mount WSIs of RP sections, this would enable quantitative reporting of tumor size, location, and grade. This would yield quantitative clinical pathology reporting and would benefit research studies, including imaging validation studies, which require an annotated histologic gold standard^12–14^. However, such manual contouring is too time consuming to perform as part of a routine clinical workflow, and is resource-intensive when performed as part of research studies. There is therefore an unmet need for an approach that can detect and grade cancerous regions accurately and quickly on digitized whole-mount histopathology images of RP tissue sections.

Many published methods have demonstrated the potential of machine learning approaches for automatic prostate cancer detection and grading on digital histopathology images^15^. High-resolution digital histopathology images acquired from RP specimens contain a large number of pixels; for instance, a typical whole-mount image of the mid-gland can contain more than four gigapixels. Consequently, most published work performs validation using a small subset of selected regions of interest (ROIs) to reduce computational demands^15^. A few studies^16–22^ have worked on cancer detection using whole-slide-images (WSIs). Doyle *et al*.^16^ and Litjens *et al*.^17,18^ have demonstrated the ability to process WSIs of much smaller biopsy tissues for finding prostate cancer using automatic systems. Monaco *et al*.^19^ and Rashid *et al*.^22^ have demonstrated cancer detection systems for finding prostate cancer on WSIs of RP tissue sections with practical processing times by classifying segmented glands, but they reported limitations regarding detection of high-grade cancer tissue using their methods. DiFranco *et al*.^20^ and Nguyen *et al*.^21^ have tested their methods on the WSIs of RP tissue sections, but the sample sizes were 14 patients and 11 WSIs for the two studies, respectively. For grading, Nir *et al*.^23^ validated on the largest number of tissue samples from tissue micro arrays (TMAs) of RP tissue sections.

In recent years, deep learning has demonstrated potential for analyzing digital histology images of prostate tissue, and many studies^18,24–28^ analyzed WSIs with larger sample sizes, compared to the earlier works mentioned above, using deep learning approach for PCa detection and grading. For PCa detection, Litjens *et al*.^18^ and Campanella *et al*.^24^ analyzed WSIs of biopsy tissues. For PCa grading, Bulten *et al*.^27^ and Ström *et al*.^28^ graded PCa on WSIs of biopsy tissues. They provided slide-level evaluations validated against pathologists’ assessments and showed that system performances were within the inter-observer variability among expert pathologists. Most^24,26–28^ of those studies focused on developing and validating systems for slide-level PCa detection or grading using biopsy tissues. Those methods provided important insight for translation to potential clinical use in diagnosis. In contrast, systems for region-level mapping and grading cancer on RP sections support post-surgical clinical decisions for follow-up treatment. Although several studies^18,25,29–31^ focused on region-level mapping, those studies did not use WSIs of RP sections. Nagpal *et al*.^26^ proposed a deep learning pipeline for Gleason scoring of RP tissue sections at the WSI level. They validated their method on an external data set containing 331 WSIs of RP tissue sections, and reported a mean diagnostic accuracy of 0.70, as compared to a mean diagnostic accuracy of 0.61 across 29 pathologists. They also reported a region-level accuracy of 97% for cancer vs. non-cancer classification, and 88% for classifying Gleason patterns of G3, G4 and G5, validating on the concordant regions across 3 pathologists on 79 WSIs. The sample sizes of each tissue type of these concordant regions were not given for the reported accuracies, limiting the interpretation of the reported region-level performance.

Comprehensive validation using all available tissue covering all clinically relevant grade groups avoids bias due to ROI selection and tests the system against the full variability in terms of staining and cancerous tissue appearance. It is also important to ensure that in cross-validation, samples are chosen such that the training and testing sets do not contain samples from the same patient^32^. This is particularly important considering 1) the heterogeneous patterns of each grade^3^, 2) the similar patterns among different grades, 3) the large staining variability among WSIs^33,34^, and 4) the requirement for practical processing times for clinical translation to the pathology laboratory.

In addition, deep learning approaches lack transparency and are challenging to interpret. Tackling this issue may be needed for widespread acceptance and regulatory approval^35^. In previous studies, semantic features (i.e. higher-level tissue components such as nuclei, lumina, etc.) have been demonstrated as crucial factors for finding and grading prostate cancer as they reflect the differentiation of cancerous tissue^36^. Many studies used features extracted from semantic feature maps and reported promising results^15^. However, the importance and applicability of those methods were not fully evaluated due to lack of comprehensive comparisons of system performance for detecting and grading PCa, especially validating on mid-gland whole-mount WSIs of RP sections.

In this study, we investigated the utility of tissue components (specifically, nuclei, lumina, and stroma/other tissue) as cues used in 7 different machine learning approaches (3 non-deep learning and 4 deep learning) for finding and grading prostate cancer on whole-mount WSIs of RP sections using 299 whole-mount WSIs from 71 RP patients.

**Figure 1:**
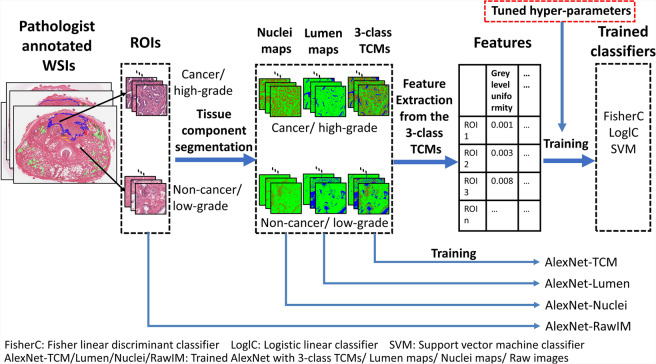
Pipeline for system training for cancer vs. non-cancer classification or high- vs. low-grade classification. For tissue component maps, nuclei are labeled in red, luminal regions are labeled in blue, and stroma/other are labeled in green.

**Figure 2:**
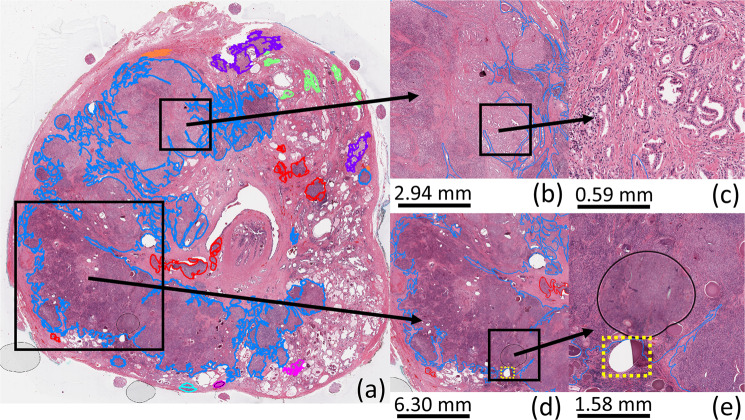
WSI of H&E stained histology prostate tissue. (**b–e**) are zoomed from the black square highlighted regions from the WSI. (**d**,**e**) show a region of torn tissue (yellow dashed square) and a region of poor focus (circle). Contour colour code: orange (G3), pink (G4), cyan (G5), red (G4 + 5), blue (G5 + 4), purple (G4 + 3), green (PIN).

Of the 299 whole-mount WSIs of mid-gland tissue sections obtained from 71 RP surgical specimens using a standard protocol at our local center^36^, 286 WSIs from 68 patients were used for validation using leave-one-patient-out (LOPO) cross-validation (CV) using all available ROIs covering each WSI (i.e. 358800 mm^2^ of tissue in total) and the remaining 13 WSIs from 3 patients were used for system tuning. After digitization, each WSI was annotated at 20X by our trained physician with each tumor contoured and the grade indicated by contour color (Figs. 1 and 2). The annotations were verified by one of two genitourinary pathologists. Each WSI was partitioned into a set of ROIs with sizes of 480 µm × 480 µm.

Figure 1 describes the training of the system. We assigned each ROI a tissue type label (i.e. cancer or non-cancer, and the Gleason grade for cancerous ROIs) based on the manual annotations done by the expert. We labeled each image pixel as one of three classes: nuclei, lumina, and stroma/other using our previously proposed method^37^, to generate three-class tissue component maps (TCMs). We also used the same technique to generate simpler binary maps: nuclei maps and lumina maps. We trained the system with 7 different machine learning approaches, enumerated as follows: 3 conventional machine learning approaches: (1) a Fisher linear discriminant classifier (FisherC), (2) a logistic linear classifier (LoglC), (3) a support vector machine classifier (SVM) with calculated texture features extracted from the TCMs, and 4 deep learning approaches via fine-tuning of AlexNet^38^ with the (4) nuclei maps (AlexNet-Nuclei), (5) lumina maps (AlexNet-Lumina), (6) three-class TCMs (AlexNet-TCM), and (7) raw image ROIs (AlexNet-RawIM).

We also masked the raw images using the TCMs to generate raw images with: (1) masked-out nuclei (i.e. the raw images are unchanged except that all nucleus regions have been changed to zero; denoted as AlexNet-Masked nuclei), (2) masked-out lumina (AlexNet-Masked lumina), and (3) masked-out nuclei and lumina (AlexNet-Masked nuclei + lumina). We repeated experiments 2 and 3 using these masked raw images. The results are reported in the supplementary materials section.

We performed 3 experiments for our cancer detection and grading problems: classifying all relevant ROIs as 1) cancer vs. non-cancer, 2) high-(G4) vs. low-(G3) grade cancer, 3) high-(G4 & G5) (i.e., G4, G5, G4 + 5, G5 + 4) vs. low-(G3) grade cancer. For experiment 2), ROIs containing ≥50% G4 cancer were considered as high-grade, and ≥50% G3 as low-grade. For experiment 3), ROIs containing ≥50% G4 and G5-involved (i.e., G4, G4 + 5, G5, G5 + 4), denoted as G4 & G5, cancer were considered as high-grade, and ≥50% G3 as low-grade. Since G4 + 3 and G3 + 4 cancer have both high- and low-grade cancer tissue, we used those tissue samples for cancer detection but not for grading experiments. The validations were conducted using all available ROIs for each WSIs using LOPO CV, during which training and testing ROIs were never drawn from the same patient. We measured cumulative error metrics of error rate, false negative rate (FNR), false positive rate (FPR), and area under the receiver operating characteristic curve (AUC), comparing the predicted label from each machine learning technique for each ROI with the reference standard label assigned to the ROI based on the pathologist’s annotations. We also measured the error rate for each tissue type separately using each of our seven approaches. Our implementation used Matlab 2018a (The Mathworks, Natick, MA), OpenCV 3.1 for SVM implementation, and PRtools 5.0 (Delft Pattern Recognition Research, Delft, The Netherlands) for implementation of FisherC and LoglC machine learning algorithms.

## Results

### Prostate cancer detection

The quantitative results for cancer vs. non-cancer classification from our LOPO CV using each method are reported in Table 1. All methods yielded AUCs higher than 0.92 except AlexNet-Lumina, which has an AUC of 0.896. AlexNet-TCM yielded the highest AUC of 0.964 (bolded in Table 1). AlexNet-RawIM and AlexNet-Nuclei yielded the second- and third-highest AUCs of 0.957 and 0.93 respectively. In general, the methods of fine-tuning AlexNet have higher AUCs and much lower FPR than the conventional machine learning methods.

**Table 1:** Cumulative error metrics for ROIs (480 µm × 480 µm) for cancer vs. non-cancer and high-vs. low-grade cancer classifications from LOPO CV.

	Cancer vs. non-cancer				G4 vs. G3				G4 & G5 vs. G3			
	Error rate	FNR	FPR	AUC	Error Rate	FNR	FPR	AUC	Error Rate	FNR	FPR	AUC
FisherC	13.5%	14.9%	13.5%	0.927	20.4%	26.2%	18.9%	0.858	20.4%	33.1%	9.3%	0.886
LoglC	12.2%	16.9%	12.0%	0.926	20.0%	27.0%	18.2%	0.850	20.9%	32.6%	10.8%	0.875
SVM	8.6%	19.9%	8.2%	0.928	21.9%	38.0%	17.8%	0.783	26.2%	43.9%	10.8%	0.815
AlexNet-RawIM	5.9%	19.2%	5.5%	0.957	11.4%	24.0%	8.2%	**0.934**	16.9%	27.4%	7.6%	0.916
AlexNet-TCM	6.1%	15.1%	5.8%	**0.964**	12.7%	28.9%	8.6%	0.904	13.2%	20.0%	7.3%	**0.923**
AlexNet-Nuclei	9.0%	15.8%	8.8%	0.937	13.9%	32.3%	9.2%	0.891	15.2%	19.8%	11.2%	0.919
AlexNet-Lumia	13.2%	21.0%	13.0%	0.896	25.9%	61.8%	16.7%	0.654	35.3%	52.9%	20.1%	0.660

G4 vs. G3: high-(G4) vs. low-(G3) grade classification. G4 & G5 vs. G3: high-(G4 & G5) vs. low-(G3) grade classification. Bolded number: highest AUC in the experiment across 7 different methods.

**Figure 3:**
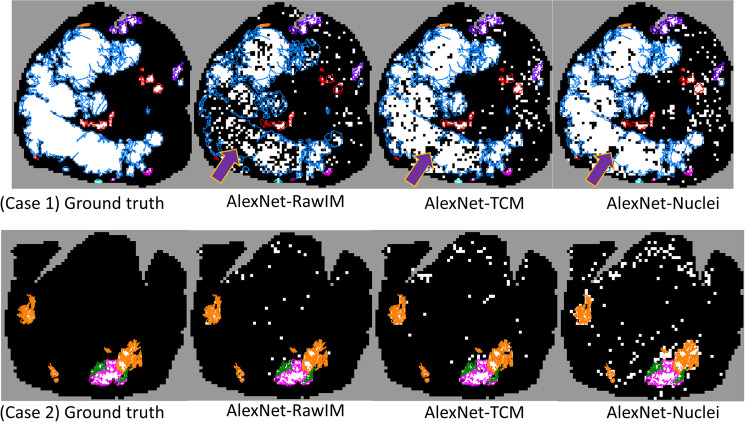
Cancer maps generated by each of the trained systems. White: cancerous tissue regions. Black: non-cancerous tissue regions. Color contours: pathologist manual annotations. The purple arrows point to unfocused areas and areas with torn tissue as indicated in Fig. 2(d,e). Contour colour code: orange (G3), pink (G4), cyan (G5), red (G4 + 5), blue (G5 + 4), purple (G4 + 3).

Figure 3 shows our system’s mapping of cancer throughout entire WSIs for two sample cases. The major cancerous and non-cancerous regions were correctly labeled by the systems for both cases. In case 1, AlexNet-TCM and AlexNet-Nuclei have similar results, while AlexNet-RawIM performs the worst, with many more false negatives in the G5 + 4 cancerous region. Figure 2 shows the original H&E stained WSI of case 1. The bottom two images are the zoomed in view from the square highlighted region from the WSI. It includes an unfocused region, and a region with torn tissue (yellow dashed square highlighted region in Fig. 2(e)). For those regions, all methods falsely classified them as negatives (regions indicated by purple arrows in Fig. 3 (case 1)). In case 2, AlexNet-RawIM and AlexNet-TCM have similar results, while AlexNet-Nuclei has more false positives. The major cancerous regions are G3, G3 + 4, and G4.

**Figure 4:**
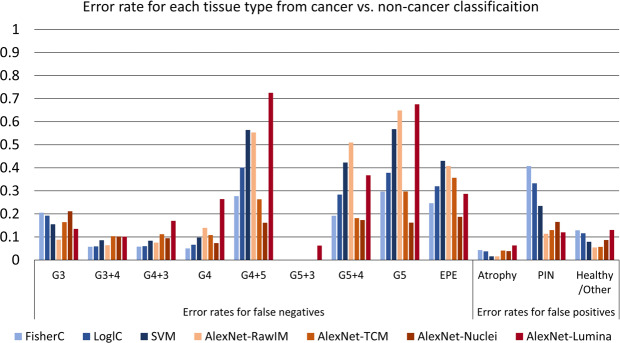
FNR for cancer tissue types, and FPR for non-cancer tissue types to reflect the error rate for each tissue type, for each classifier from leave-one-patient-out cross-validation of cancer vs. non-cancer classification.

We calculated the FNRs for cancerous ROIs and the FPRs for non-cancerous ROIs, effectively computing the error rates for each of these tissue types. The results of the LOPO CV experiments are shown in Fig. 4. Table 2 shows the number of ROIs used for each tissue type. G5, G4 + 5, G5 + 4, and EPE yielded higher error rates. Table 2 and Fig. 4 demonstrate that in general, higher error rates corresponded to smaller sample sizes; G5 + 4 was the exception. For those tissue types, with the exception of EPE, AlexNet-Nuclei, AlexNet-TCM, and FisherC yielded much lower error rates than the other methods. Among those methods, AlexNet-Nuclei had the lowest error rate. For G4-involved tissue types (i.e. G3 + 4, G4 + 3, and G4), FisherC yielded the lowest error rates, and LoglC achieved similar performance. For other tissue types, AlexNet-RawIM yielded the lowest error rates. Those tissue types are primarily non-cancerous and G3 cancerous tissues, and they have larger sample sizes.

**Table 2:** Number of ROIs for each tissue type.

Cancerous ROIs										Non-cancerous ROIs		
Tissue types	G3	G3 + 4	G4 + 3	G4	G4 + 5	G5 + 4	G5	G5 + 3	EPE	Atrophy	PIN	Healthy tissue/BPH
Sample size	14719	6008	3839	3949	725	8216	37	16	272	5433	26449	1178814

### Prostate cancer grading (high- vs. low- grade)

The quantitative results for high- vs. low-grade cancer classification from our LOPO CV using each method are reported in Table 1. For high-(G4) vs. low-(G3) grade classification, AlexNet-RawIM yielded the highest AUC of 0.934, followed by AlexNet-TCM and AlexNet-Nuclei with AUCs of 0.904, and 0.891 respectively. For high-(G4 & G5) vs. low-(G3) grade classification, AlexNet-TCM, AlexNet-Nuclei and AlexNet-RawIM are the top three performing methods with AUCs of 0.923, 0.919, and 0.916 respectively. SVM and AlexNet-Lumina had much lower AUCs than other methods for both of the experiments. Except for AlexNet-Lumina, methods of fine-tuning AlexNet yielded higher AUCs, lower FPRs and FNRs than the conventional machine learning approaches for both experiments.

**Figure 5:**
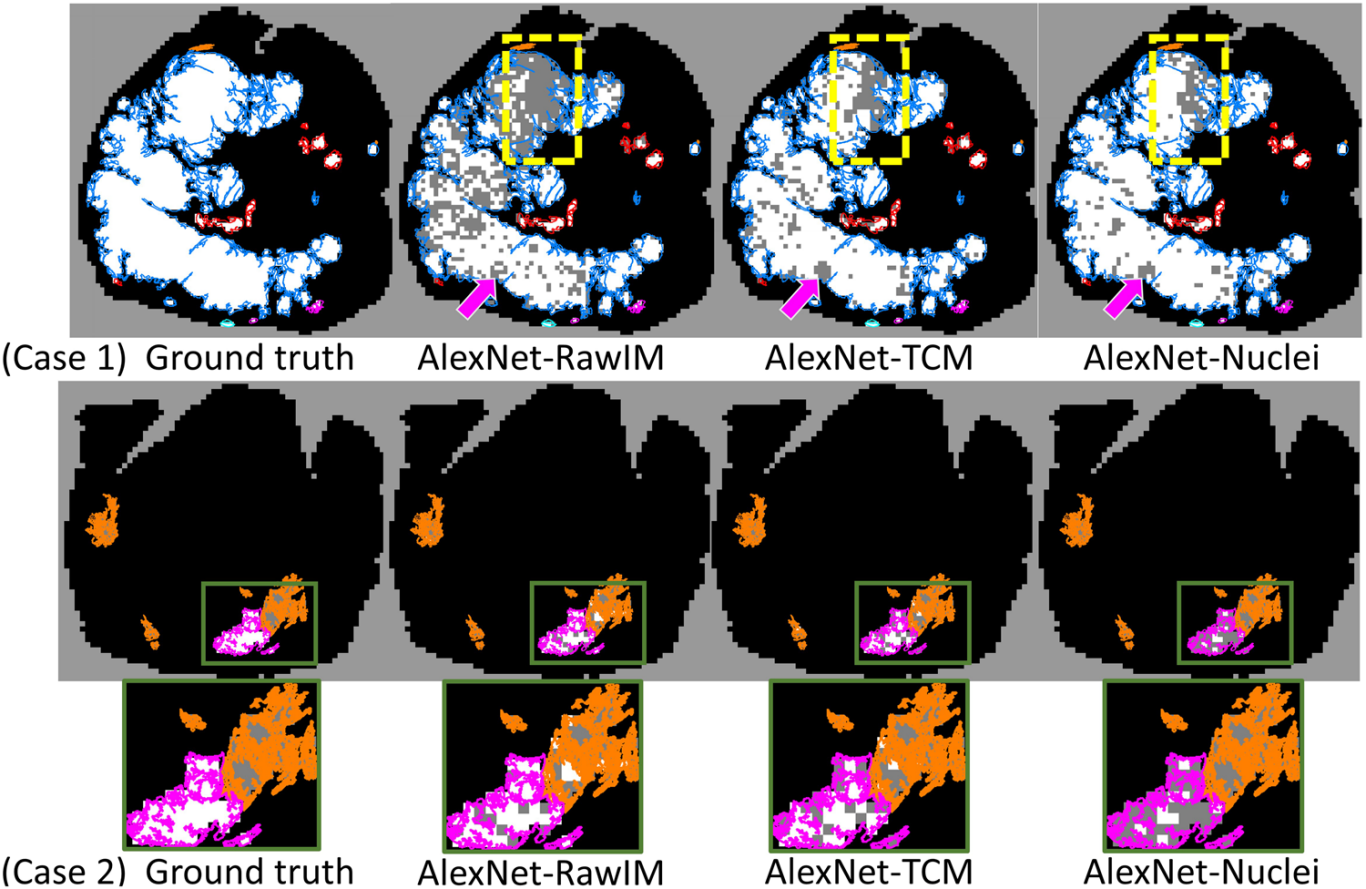
Label maps for high- vs. low-grade cancer grading generated by each of the trained systems. White: high-grade cancerous tissue regions. Grey: low-grade cancerous tissue regions. Black: tissue section. Color contours: pathologist’s manual annotations. The region highlighted by the yellow square refers to the tissue regions in Fig. 2(b,c). The region indicated by the pink arrow refers to the unfocused areas and regions with torn tissue in Fig. 2(d,e). Contour colour code: orange (G3), pink (G4), cyan (G5), red (G4 + 5), blue (G5 + 4).

In Fig. 5, two samples of whole-slide mapping of graded cancer are shown. The major cancerous regions are correctly graded and labeled by the systems. For case 1, similar to cancer detection, AlexNet-TCM and AlexNet-Nuclei yielded similar performance, and AlexNet-RawIM had many more false negatives. For the unfocused and torn tissue regions (Fig. 2(d,e)) and the regions with lower Gleason patterns (Fig. 2(b,c)), all methods incorrectly labeled the tissue as negative (regions highlighted with a yellow dashed square in Fig. 5). In case 2, AlexNet-TCM and AlexNet–RawIM yielded similar performance, while AlexNet-Nuclei had more false negatives (regions highlighted with a green square in the zoomed in view in Fig. 5).

**Figure 6:**
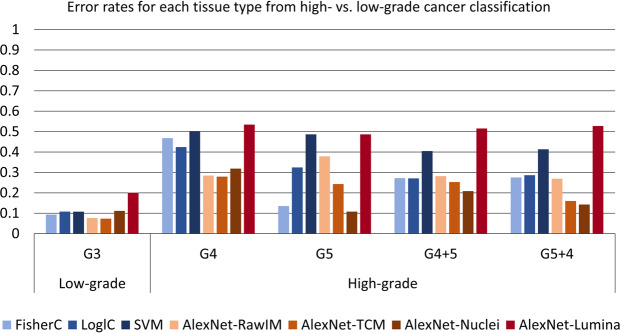
Error rate (FNR for high grade cancer, FPR for low-grade cancer) for each tissue type for each classifier from leave-one-patient-out cross-validation of high-(G4 & G5) vs. low-(G3) grade classification.

From the LOPO CV experiments for high-(G4 & G5) vs. low-(G3) classification, we calculated the error rates for each tissue type (i.e. taking high-grade cancer as “positive” in these experiments, we calculated FNRs for high-grade cancer tissues types, and the FPRs for the low-grade cancer tissue types). The results are shown in Fig. 6. We found higher error rates for each of the high-grade cancer tissue types, compared to the error rate for the low-grade cancer tissue type. For tissue types which have G5 cancer tissue involved, AlexNet-Nuclei yielded the lowest error rates. For all other tissue types, AlexNet-TCM had the lowest error rates.

For the purpose of comparison, the results including the use of the raw images masked according to the TCMs are reported in Supplementary Table S3 (results for overall performance) and Supplementary Fig. S1 (the error rates for each tissue type).

## Discussion

Although using machine learning to analyze H&E histology images for prostate cancer detection and grading is an active research field, there are relatively few studies validating on whole-mount RP tissue sections, and the use of deep learning for this problem is still relatively new^18^. In addition, many studies have demonstrated that tissue component features are important for prostate cancer detection and grading^15^, but the effects of those tissue components on system performance for cancer detection and grading for different types of tissue were not directly compared. Therefore, in our study we used different machine learning approaches with different tissue component maps, and compared the performances for both the cancer detection and grading problems on the largest expert-annotated dataset of RP tissue sections reported thus far.

In general, for both cancer detection and grading, AlexNet-TCM achieved the best overall performance, followed closely by AlexNet-RawIM. Conventional machine learning approaches demonstrated inferior but comparable overall performance (AUCs in Table 1). This suggests that the 3-class TCMs provide a set of major cues for prostate cancer detection and grading. This is also reflected by very similar performance of AlexNet-RawIM to that of AlexNet-TCM. The observed slightly inferior overall performance by using raw images could be due to irrelevant or redundant information (e.g. red blood cells) from the raw images, resulting in confounders to which the network could overfit.

With the exception of AlexNet-Lumina, fine-tuning AlexNet based approaches achieved better performance than the conventional machine learning based approaches for both cancer detection and grading. This suggests fine-tuning AlexNet outperforms conventional machine learning based approaches overall. This can also be supported by direct comparison of AlexNet-TCM and conventional machine learning approaches. For cancer detection, AlexNet-TCM had lower error rates for most tissue types (Fig. 4), therefore better overall performance was achieved. For grading, it had higher AUCs for the two grading experiments (Table 1). For the second experiment, with the exception of G5-involved cancer, AlexNet-TCM had lower error rates for all tissue types (Fig. 6). The worst performance was yielded using AlexNet-Lumina, suggesting that the lumina maps provide insufficient information for our problem. This is also suggested by the much larger performance differences between AlexNet-Lumina and other methods for cancer grading, compared to the performance differences for cancer detection (Table 1). We speculate that because the tissue appearances are much more similar for cancerous tissues of different grades than for cancer vs. non-cancerous tissues, more tissue information is needed for cancer grading.

The performances of all the machine learning methods we used are sensitive to sample size, with sensitivity varying according to machine learning method used, classification task, and tissue type. Lower error rates are usually associated with larger sample sizes, and vice versa, except for G5 + 4 cancer tissue (Table 2, Figs. 4 and 6). G5 + 4 appears to be an exception with a sample size of 8,216, which is relatively large. However, most of the G5 involved cancer (including G5 + 4) occurred in a small number of patients. Since we used LOPO CV, tissue from a single patient never appeared in both the training and testing sets, reducing the number of occurrences of G5 cancer in training. For cancer detection, AlexNet-RawIM was the most sensitive to the sample size, while AlexNet-TCM and AlexNet-Nuclei, and the conventional machine learning approaches (except for SVM) were less sensitive to sample size (Fig. 4 and Table 2). This suggests higher-order semantic features (e.g. tissue component based features) can improve the robustness of the system to smaller training sample sizes. However, for cancer grading, this was not the case for G4 cancer tissues (Fig. 6), where conventional machine learning based approaches had substantially higher error rates than the AlexNet-based approaches, compared to other tissue types. We speculate that this could be due to the relative similarity of the G3 and G4 patterns, requiring more complex deep learning model to differentiate them.

For both cancer detection and grading, AlexNet-Nuclei achieved similar but slightly inferior overall performance to AlexNet-TCM and AlexNet-RawIM (Table 1), and the best performance for high-grade cancer tissue types (Figs. 5 and 6). This suggests that among the 3-class TCMs, the nuclei maps capture the key cues for our problems, especially for higher-grade cancer tissue types (i.e. G5, G4 + 5, G5 + 4). Adding lumina features (using AlexNet-TCM) or other features (using AlexNet-RawIM) improved the performance for most tissue types, but not for higher-grade cancer tissue types (Figs. 5 and 6). This can also be evidenced by the superior performance in experiment 2 and the inferior performance in experiment 3 by AlexNet-Masked nuclei+lumina than AlexNet-TCM (Supplementary Table S3). We speculate that this is because providing tissue images of stroma/other information may weight the features extracted them much more than that was weighted in TCMs (i.e. labeled as a single class tissue type), which reduced the weights of features extracted from the labeled nuclei, thus resulting to inferior performance for identifying G5-involved tissue types (Supplementary Fig. S1). Identifying key features within the stroma/other tissue class may improve the overall performance for grading by further segmenting tissue components (e.g. stroma, cytoplasm). This also makes sense from the clinical pathology perspective. Since higher grade cancer tissue (G5-involved cancer tissues) are poorly differentiated with merged glands and much less stroma tissue (Fig. 2), luminal and stroma features are not helpful for identifying those tissue types. Also, those tissues have larger amounts of nuclei, which leads to darker hematoxylin stain (Fig. 2). Thus, the 3-class TCMs and raw images are likely to contain more extraneous information, compared to the nuclei maps. Vice-versa, this explains better performance for the G3-involved and non-cancerous tissue types (Figs. 5 and 6, and Table 1, G4 vs. G3) using raw images, and consistent performance across all tissue types using TCMs.

Kwak *et al*.^29^ have also previously reported that a nucleus seed map is essential for prostate cancer detection using machine learning techniques. On a data set consisting of 707 sample cores from 4 TMAs, they found that nucleus seed maps trained with their proposed convolutional neural network (CNN) yielded better performance than raw images trained with other CNNs (including AlexNet). The use of different data sets and sample sizes in their study may explain the differences with respect to our results.

Nagpal *et al*.^26^ also proposed a deep learning pipeline and validated it with large data sets from multiple institutions, reviewed by multiple pathologists. Their paper and ours are complementary in the sense that their approach focused primarily on Gleason scoring at the slide level (i.e. determining one Gleason score for the whole slide), whereas our study focused on region-level mapping (i.e. determining one Gleason grade for each ROI). They reported a secondary region-level analysis on 79 WSIs; the number of patients from which these WSIs were drawn was not reported. They measured their deep learning system’s accuracy as compared to the regions of those WSIs where all three of their pathologists were concordant in their regional assessments; the total area and grade distribution of these concordant regions were not reported. Their reported concordance rate between their deep learning system’s region-level assessments and their pathologists’ assessments was 97% for cancer vs. non-cancer classification, and 88% for Gleason pattern classification. It is important to interpret these results with the consideration that more potentially challenging regions on the slides, where pathologists’ assessments were discordant, were excluded in computing these concordance values. Interpretation of these results would also be helped by knowledge of the distribution of positive vs. negative and Gleason pattern labels, to understand the impact of any label imbalance on the data set. It would be an interesting avenue of future work to test their proposed deep learning system for region-level classification throughout all ROIs on all WSIs in the data set.

The results of this study must be interpreted in the context of its limitations. First, all of the tissue sections were processed in one clinical pathology facility. Since tissue processing conditions and protocols vary from center to center, multi-center studies are needed to translate these techniques to practice. We would expect these issues to affect the methods using raw images more than those using TCMs, the computation of which is adaptive and calibration-free. Second, since our study was validated using annotations made by one physician and verified by one of two genitourinary pathologists, measurement of the impact of inter-pathologist assessment variability not within the scope of this study. Third, our conventional machine learning methods may yield sub-optimal performance due to the following reasons: 1) we only investigated first- and second-order statistical features for texture feature quantization, 2) backward feature selection is a greedy algorithm, and 3) there exist many types of classifiers that were not tested in our study. Fourth, since our validation is at the ROI level and our pathologist’s annotations were provided at the tumor level, we were unable to provide labels to ROIs from the G3 + 4 and G4 + 3 tumor regions, which have mixed high- and low-grade Gleason patterns. Fifth, Gleason scoring at WSI-level and the patient-level are not included in our reference standard, and thus our analysis is limited to the ROI level. Although it stands to reason that performance of the method at the WSI- and patient-level would be concordant with measured performance at the ROI level, it would be important to verify this in future work.

In conclusion, this work demonstrated automatic prostate cancer detection and grading on gigapixel WSIs of RP tissue sections using machine learning approaches with the state-of-the-art performance and practical processing time, and testing on the largest amount of expert annotated tissue so far. Fine-tuning pre-trained AlexNet demonstrated better performance than conventional machine learning based approaches overall. We found that the 3-class TCMs captured the main information for both prostate cancer detection and grading, and yielded robust performance across different tissue types and sample sizes. The best overall performance was achieved using the 3-class TCMs with transfer learning using AlexNet. In the 3-class TCMs, the nuclei maps provided the most important information overall, and were essential for classifying G5-involved cancerous tissue types for both cancer detection and grading. Future work could include detection and quantification of tissue margin involvement and other prognostic pathology features.

## Methods

### Data

#### Materials and imaging

We obtained informed consent from all 71 patients in our study, and this study was approved by our institutional Health Sciences Research Ethics Board. All experiments were performed in accordance with relevant guidelines and regulations. All patients had biopsy-confirmed prostate cancer, clinical stage T1 or T2. From these patients we obtained 299 H&E-stained, 4 μm thick, paraffin-embedded mid-gland tissue sections, and acquired a whole-slide image from each. We used the same protocol as described in our previous paper^36^ and processed all tissues in our clinical pathology laboratory. We used two different scanners to obtain images at 20× (0.5 μm/pixel) in bigtiff pyramid format without compression: an Aperio ScanScope GL (Leica Biosystems, Wetzlar, Germany) for sections from 46 patients and an Aperio ScanScopeAT Turbo (Leica Biosystems, Wetzlar, Germany) for sections from the other 25 patients. This process yielded 24-bit RGB color images at 0.5 μm/pixel.

#### Manual annotation

A trained physician (Gaed) contoured and graded each WSI at 20× magnification using a Cintiq 12WX pen-enabled display (Wacom Co. Ltd., Saitama, Japan) with the ScanScope ImageScope v11.0.2.725 image viewing software (Aperio Technologies, Vista, CA, USA)^3^. Each contour was verified, and edited as necessary, by one of two genitourinary pathologists (Moussa or Gomez). The zoomed region in Fig. 2 demonstrates the level of precision of our contouring.

#### Ground truth ROI labeling

We separated each WSI into a set of square 960 × 960 pixel ROIs. We assigned each ROI a label according to the manual pathology annotations with 50% threshold. For cancer detection, ROIs containing more than 50% cancerous tissue were considered cancerous; all other ROIs were considered non-cancerous. Non-cancerous regions contained confounders such as atrophy, benign prostatic hyperplasia (BPH), high-grade prostate intraepithelial neoplasia (PIN), and inflammation. For cancer grading, ROIs containing more than 50% high-grade cancer tissue were considered positive, otherwise negative. The sample size of each tissue type is shown in Table 2.

### Data separation for system tuning and feature selection

We used a “tuning data set” of 13 WSIs from 3 patients for hyper-parameter tuning and feature selection. The tuning data set was entirely separate from the rest of the data and was not used for cross validation. We used the 68 remaining patients for cross-validation. WSIs from both scanners were included in both the tuning and cross-validation data sets.

### Tissue component mapping

Tissue staining makes salient tissue components that have semantic meaning to the pathologist. We used our previously developed methods^37^ to assign a label to each image pixel to generate (1) a nuclei map, (2) a lumina map, (3) a 3-class TCM for further analysis, via (1) segmentation of nuclei using color deconvolution^39^ and our previously proposed adaptive thresholding algorithm^37^ to generate the nuclei map, (2) segmenting luminal areas by global thresholding in the red-green-blue (RGB) color space to generate the lumina map, and (3) combining the results of nuclei and lumina segmentation and designating the rest pixels as “other” to generate the 3-class TCM. The details of these methods are described as follows.

#### Nucleus mapping

We separated the H&E stains into three image channels of hematoxylin stain, eosin stain, and the background, using a color deconvolution algorithm^39^. We applied this algorithm to each ROI independently using the standard deconvolution matrix used by Ruifrok and Johnston^39^, which separated each ROI into three grey-level images corresponding to the amount of hematoxylin, eosin, and background respectively. Most substances within nuclei bind to hematoxylin since they are basophilic. Therefore we used the hematoxylin channel for nuclei segmentation by adaptive thresholding^37^.

There are large staining differences across different images from different patients even if the tissue sectioning, staining and scanning were performed using consistent protocols in the same laboratory. The grey-level intensity variation in the hematoxylin channel across different WSIs, which results from staining variability, makes global thresholding not applicable for nucleus segmentation. We therefore used our previously proposed adaptive thresholding method^37^. For each WSI, the segmentation threshold was selected based on a cumulative assessment of 2,000 randomly-selected 120 *µm* × 120 *µm* ROIs to lie within the prostate (i.e. to avoid clear slide areas) and to not contain tissue marking dye (i.e. to avoid areas of artefact). This makes the segmentation threshold specific to each WSI of the RP tissue section, therefore compensating for the staining variability across different WSIs. The thresholds for sample selection were derived by inspection of the tuning data set only.

This process will sometimes mislabel RBCs as nuclei because, like nuclei, RBCs stain with hematoxylin. We can distinguish RBCs from nuclei based on the fact that RBCs have higher red-pink saturation. We computed a cumulative histogram from 100 40 *µm* × 40 *µm* RBC ROIs from our tuning data set and used hue-saturation-intensity thresholds (hue ≥ 0.95/1, saturation ≥0.72/1, and intensity ≥0.6/1). After morphological dilation with a disk-shaped structuring element of radius = 4 *µm* (approximate radius of human red blood cells), we obtained an RBC mask and subtracted it from the nucleus map to eliminate false RBCs^37^.

#### Lumina mapping

Lumen is typically nearly white on microscopy images. We therefore thresholded luminal pixels with values of red ≥ 0.86/1, green ≥ 0.71/1, and blue ≥ 0.82/1, using the same approach as described above (cumulative histogram based on the tuning data set).

### Tuning ROI size and down-sampling ratio

Experimenting with our tuning data set, we selected a nearest-neighbor down-sampling ratio of 0.25, and an ROI size of 480 *µm* × 480 *µm* (960 *pixels* × 960 *pixels*). We ranked cancer detection performance according to the area under the receiver operating characteristic curve using FisherC with all features in a leave-one-patient-out cross-validation scheme to select these parameters^37^. These parameters were unchanged for all experiments in this paper.

### Feature extraction and selection

24 first-order and 132 second-order statistical features were calculated from the TCM of each ROI, giving a total of 156 features. The second-order statistical features were calculated from the grey-level co-occurrence matrix (GLCM)^40^ and grey-level run length matrix (GLRLM)^41^. GLCMs and GLRLMs were calculated using neighbors in four directions without aggregation ([(0,1) represents direction 1 in the Supplementary Table S1, (−1,1) represents 2, (−1,0) represents 3, and (−1,−1) represents 4]). 22 different GLCM (neighbor distance = 1) and 11 GLRLM features were calculated. We calculated a total of 156 features; (22 GLCM + 11 GLRLM) × 4 directions + 24 first-order features = 156.

For cancer vs. non-cancer classification, the 14 top-ranked features were selected from the calculated feature set of 156 features using backward feature selection on the tuning dataset, which selects the features by ranking the AUCs from the LOPO CVs using a Fisher linear classifier. The chosen texture features are listed in Supplementary Table S1. For high- vs. low-grade cancer classification, we selected the 41 top ranked features by using the same feature selection method for cancer vs. non-cancer classification with the tuning dataset of high-(G4) and low-(G3) grade cancer samples. The chosen features were used for both G4 vs. G3, and G4 & G5 vs. G3 grading experiments. The chosen texture features are listed in Supplementary Table S2.

### Cancer detection and grading using machine learning

For prostate cancer detection, we classified each ROI as cancerous vs. non-cancerous using the 14 selected features calculated from the 3-class TCMs. We performed supervised machine learning using (1) a Fisher’s least square linear discriminant classifier, (2) a logistic linear classifier, and (3) a NU-SVM with a radial basis function kernel (parameters tuned as cost = 12.5, and gamma = 0.50625 using our tuning dataset). Each of these approaches is denoted as follows throughout the paper: (1) FisherC, (2) LoglC, and (3) SVM, respectively.

For cancer grading, we classified each ROI of all relevant cancerous regions as high- vs. low-grade by two experiments: (1) all cancerous regions of G4 and G3 for high-(G4) vs. low-(G3) classification; (2) all cancerous regions of G4 & G5 and G3 for high-(G4 & G5) vs. low-(G3) grading); using the 44 selected features calculated from the 3-class TCMs. Similarly as for cancer detection, we performed supervised machine learning using (1) FisherC, (2) LoglC, (3) SVM (a C-SVC with a linear kernel with parameters tuned as cost = 2.7, and gamma = 0.03375 by tuning using high-(G4) and low-(G3) grade samples from the tuning data set).

For both cancer detection and grading, we also used transfer learning via fine-tuning AlexNet with our nuclei maps, lumina maps, 3-class TCMs and raw image ROIs, denoted as: AlexNet-Nuclei, AlexNet-Lumina, AlexNet-TCMs, and AlexNet-RawIM, respectively. AlexNet was trained using 1.2 million non-medical images from the ImageNet LSVRC-2010 challenge^38^. The final fully connected layer of AlexNet was replaced by a fully connected layer with a 2-way output followed by the 2-way softmax algorithm with a 2-class label output for each of our experiments ((1) cancerous vs. non-cancerous for cancer detection, (2) high- (G4) vs. low-grade (G3), and (3) high-(G4 & G5) vs. low-(G3) grade for cancer grading). We used cross-entropy as our loss function. We used random numbers to initialize the weights and biases of the replaced layers. For all other layers, we set the initial learning rate to α = 0.0001, and α = 0.002 for the output layer. For gradient descent, we used the adaptive moment estimation (‘Adam’) optimizer^42^, with *β*_1_ = 0.9, *β*_2_ = 0.999, and ε=10^−8^ ^42^. We used our tuning data set to set mini-batch size = 200, maximum epoch = 10.

We used nuclei maps, lumina maps, and 3-class TCMs as input images to fine-tune pre-trained AlexNet respectively for each of the 3 experiments. These maps were converted into RGB color images, such that the nuclei, stroma/other tissue, and lumina are labeled in red, green, and blue respectively. We resized each ROI with size of 240 × 240 × 3 to 227 × 227 × 3 to conform to the standard input size for AlexNet by using bilinear interpolation. We repeated the experiment using the same method with the “raw” unmodified H&E images instead of the TCMs.

### Experiments and validation

#### Prostate cancer detection

We performed LOPO CV for each of the tested machine learning approaches. No same-patient samples were used in both the training and testing sets in any CV iteration. Our data set contains many more non-cancerous than cancerous ROIs in our data set. Consequently, during training, we performed random subsampling of the negative samples to balance the positive (cancerous) and negative (non-cancerous) samples. During testing, all tissue on all slides was classified. That is, we performed testing on all ROIs covering each WSI in our 68-patient set. The receiver operating characteristic (ROC) curve was computed using the cumulative predicted confidences from each trained system, and we calculated the AUC from the ROC. We calculated the cumulative error rate, FPR, and FNR by comparing the predicted labels (using the fixed operating point corresponding to the confidence level of 0.5 in all experiments) of each ROI to the designated ROI label based on the pathologist’s annotations, with an ROI considered positive when assessed by the pathologist to contain ≥ 50% cancer. The sample sizes for each tissue type are shown in Table 2. We also calculated the error rates (FNRs for cancerous tissue types; FPRs for non-cancerous tissue types) for each tissue type using LOPO CV.

#### Prostate cancer grading

We performed the same CV as for cancer detection, for each approach for high- vs. low-grade cancer classification. During training, we balanced the positive (high-grade) and negative (low-grade) samples by random duplication of the samples for whichever class had the smaller sample size. The validation was done by comparison to the pathologist’s annotations, with an ROI considered high-grade when assessed by the pathologist to contain ≥ 50% 1) G4 for high-(G4) vs. low-(G3) grading, and 2) G4 or G5 for high-(G4 & G5) vs. low-(G3) grading. For high-(G4 & G5) vs. low-(G3) grading, we calculated the FNRs for the high-grade cancer tissue types using LOPO CV.

### Ethical approval and informed consent

This study was approved by our institutional Health Sciences Research Ethics Board (Western University Health Sciences Research Ethics Board, London, Ontario, Canada) with the written consent obtained from all patients.

## Supplementary information

Supplementary Information.

## Data Availability

The raw data (classifier output for each ROI) are available on request, to test the reproducibility of the reported error metric values. The algorithms and methods are available from the corresponding author upon request.
