# Supplementary material for: Histologic tissue components provide major cues for machine learning-based prostate cancer detection and grading on prostatectomy specimens
Source: Sci Rep. 2020 Jun 18;10:9911. doi: 10.1038/s41598-020-66849-2 (PMC7303108; doi:10.1038/s41598-020-66849-2)
Supplement: Supplementary file 1 — Supplementary Information. [file 41598_2020_66849_MOESM1_ESM.pdf]

Histologic tissue components provide major cues for machine learning-based prostate cancer detection and grading on prostatectomy specimens

Wenchao Han[1,2,6,*], Carol Johnson[1], Mena Gaed[3], Jose A. Gomez-Lemus[3], Madeleine Moussa[3], Joseph Chin[4,5], Stephen Pautler[4,5], Glenn Bauman[2,5], and Aaron Ward[1,2,5,6,*]

[1]Baines Imaging Research Laboratory, London Regional Cancer Program, Depts. of [2]Medical Biophysics, [3]Pathology and Laboratory Medicine, [4]Surgery, [5]Oncology, Western University, [6]Lawson Health Research Institute, London, Ontario, Canada

Correspondence should be addressed to A.W. (email: aaron.ward@uwo.ca) and W.H. (email: whan25@uwo.ca)

Supplementary materials

Mean gradient value	GLCM entropy-2
GLCM IDM-2	GLCM correlation-1
GLCM IDM-3	GLCM cluster shade-3
GLRLM short run emphasis-3	GLCM IMC2-1
GLRLM short run low gray level emphasis-1	GLCM IMC2-2
GLRLM short run low gray level emphasis-3	GLCM energy-1
GLRLM short run low gray level emphasis-4	GLCM energy-2

Supplementary Table S1: 14 selected features for cancer vs. non-cancer classification. GLCM: grey level co-occurrence matrix. GLRLM: grey level run length matrix. IDM: inverse difference moment. IMC: information measure of correlation. 1, 2, 3, 4: one of the 4 directional offsets used for calculating the matrix.

Gray level bimodality coefficient	GLCM difference entropy-1
GLCM correlation-1	GLCM IMC1-3
GLCM correlation-2	GLCM IMC2-1
GLCM correlation-4	GLCM IMC2-4
GLCM variance-1	GLCM IDM-2
GLCM sum average-3	GLCM IDM-3
GLRLM short run emphasis-2	GLCM IDM-4
Proportion of stroma	GLRLM run length nonuniformity-1
Gray level variance	GLRLM run percentage-4
Gray level uniformity	GLRLM short run high gray level emphasis-2
GLCM entropy-3	GLRLM long run low gray level emphasis-1
GLCM variance-3	GLRLM long run low gray level emphasis-2
GLCM variance-4	Gray level entropy
GLCM sum average-1	GLCM cluster prominence-3
GLCM sum average-4	GLCM sum average-2
GLCM sum entropy-1	GLCM difference entropy-2
GLCM sum entropy -3	GLCM difference entropy-3
GLCM sum entropy -4	GLRLM long run emphasis-2
GLCM sum variance-1	GLRLM gray level nonuniformity-3
GLCM sum variance-3	GLRLM low gray level run emphasis-2
GLCM sum variance-4	

Supplementary Table S2: 41 selected features for high- vs. low-grade cancer classification. GLCM: grey level co-occurrence matrix. GLRLM: grey level run length matrix. IDM: inverse difference moment. IMC: information measure of correlation. 1, 2, 3, 4: one of the 4 directional offsets used for calculating the matrix.

	G4 vs. G3				G4 & G5 vs. G3			
	Error Rate	FNR	FPR	AUC	Error Rate	FNR	FPR	AUC
**FisherC**	20.4%	26.2%	18.9%	0.858	20.4%	33.1%	9.3%	0.886
**LoglC**	20.0%	27.0%	18.2%	0.850	20.9%	32.6%	10.8%	0.875
**SVM**	21.9%	38.0%	17.8%	0.783	26.2%	43.9%	10.8%	0.815
**AlexNet-RawIM**	11.4%	24.0%	8.2%	**0.934**	16.9%	27.4%	7.6%	0.916
**AlexNet-TCM**	12.7%	28.9%	8.6%	0.904	13.2%	20.0%	7.3%	**0.923**
**AlexNet-Nuclei**	13.9%	32.3%	9.2%	0.891	15.2%	19.8%	11.2%	0.919
**AlexNet-Lumina**	25.9%	61.8%	16.7%	0.654	35.3%	52.9%	20.1%	0.660
**AlexNet-Masked nuclei+lumina**	11.5%	31.2%	6.6%	0.931	16.7%	25.0%	9.5%	0.912
**AlexNet-Masked nuclei**	12.5%	31.9%	7.6%	0.914	16.9%	24.5%	10.2%	0.906
**AlexNet-Masked lumina**	10.8%	25.2%	7.2%	0.931	19.7%	33.9%	7.4%	0.899

Supplementary Table S3: Cumulative error metrics for ROIs (480μm×480μm) for high-vs. low-grade cancer classifications from LOPO CV. G4 vs. G3: high-(G4) vs. low-(G3) grade classification. G4 & G5 vs. G3: high-(G4 & G5) vs. low-(G3) grade classification. Bolded number: highest AUC in the experiment across 10 different methods.

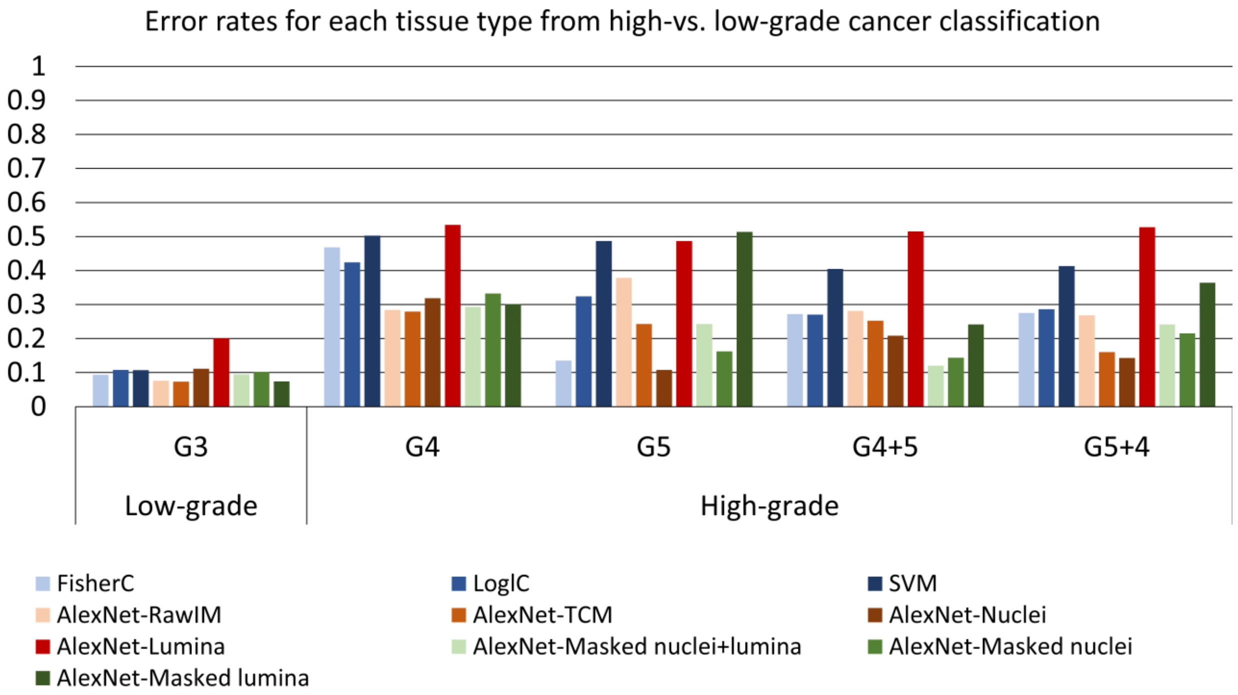

Supplementary Fig. S1: Error rate (FNR for high grade cancer, FPR for low-grade cancer)) for each tissue type for each classifier from leave-one-patient-out cross-validation of high-(G4 & G5) vs. low-(G3) grade classification.

Supplementary methods and results

The quantitative results for high- vs. low-grade cancer classification from our LOPO CV using each method (including the use of masked tissue components) are reported in Supplementary Table S3. For high-(G4 & G5) vs. low-(G3) grade classification, error rates for each tissue type were reported in in Supplementary Fig. S1.

To make masked tissue component images, we combined the TCMs and raw images by masking tissue components on the raw images with: (1) both nuclei and lumina, (2) nuclei, (3) lumina. The deep learning pipeline was performed to each types of the masked images and denoted as: (1) AlexNet-Masked nuclei+lumina, (2) AlexNet-Masked nuclei, (3) AlexNet-Masked lumina.

For high-(G4) vs. low-(G3) grade classification, AlexNet-Masked nuclei+lumina yielded better performance than AlexNet-TCM (Supplementary Table S3). For high-(G4 & G5) vs. low-(G3) grade classification, comparing to AlexNet-RawIM, AlexNet-Masked nuclei+lumina achieved similar overall performance (Supplementary Table S3). For different tissue types, AlexNet-Masked nuclei+lumina yielded similar performance for G3 and G4 tissue types, and better performance for G5 involved cancer tissue types (Supplementary Fig. S1).
